# Prevalence and predictors of female sexual dysfunction: a protocol for a systematic review

**DOI:** 10.1186/2046-4053-3-75

**Published:** 2014-07-11

**Authors:** Megan E McCool, Melissa A Theurich, Christian Apfelbacher

**Affiliations:** 1 Medical Sociology, Department of Epidemiology and Preventive Medicine, University of Regensburg, Dr. Gessler Strasse 17, 93051 Regensburg, Germany; 2 Division of Metabolic and Nutritional Medicine, Dr. von Hauner Children's Hospital, Ludwig-Maximilians-University of Munich, Lindwurmstrasse 4, 80337 Munich, Germany

**Keywords:** Systematic review, Female sexual dysfunction, Sexual disorder, Prevalence, Predictors, General population

## Abstract

**Background:**

Sexual function is an essential component of life. For this reason, sexual dysfunction can have a negative impact on the wellbeing of men and women alike. Since the turn of the 21st century, research on female sexual dysfunction (FSD) has gained momentum. While FSD is often assessed in people with ill health, sexual dysfunction is an illness of its own entity and is also prevalent in non-patient populations. A critical review of current literature on female sexual dysfunction in general populations will shed light on possible determinants as well as at-risk groups. Thus, the aim of this systematic review is to assess the prevalence and the predictors of female sexual dysfunction in general populations.

**Methods/Design:**

A systematic review of current literature on FSD will be performed. Studies will be considered for review if they report quantitative data on the prevalence of female sexual dysfunction. Outcome measures will include the prevalence of FSD, the time period assessed, and significant predictors for each domain of FSD. The scientific databases MEDLINE, EMBASE, PsycINFO, and Web of Science will be systematically searched in cooperation with a medical research librarian. Hand searches for further relevant publications will also be undertaken. Screening of search results and extraction of data from included studies will be conducted cooperatively by two authors. The quality of the studies will be appraised and documented. Results will be compiled and presented in evidence tables.

**Discussion:**

In the past decade, population-based studies on female sexual dysfunction have increased in number and grown more varied in their cultural settings. This review aims to provide a current overview of the prevalence of female sexual dysfunction in populations from various countries, cultures, and age groups in order to provide a better understanding of its effect on women's lives today.

## Background

### Introduction

Sexual function is an essential component of life. For this reason, sexual dysfunction can have a negative impact on the wellbeing of an individual. According to the Diagnostic and Statistical Manual of Mental Diseases (DSM), sexual dysfunction is characterized by a disturbance in the processes that characterize the sexual response cycle or by pain associated with sexual intercourse [1]. Among women, sexual dysfunction generally falls into four categories: hypoactive sexual desire disorder, female sexual arousal disorder, female orgasm disorder, and pain disorders [2].

Female sexual dysfunction (FSD) has attracted more interest in the past few decades [2]. At the turn of the 21st century, FSD was identified as a significant yet largely uninvestigated public health problem [3]. At the time, there was little population-based data available concerning the prevalence, predictors, and consequences of this disorder [3].

In 2004, West et al. performed a systematic review of global literature on the prevalence and predictors of FSD in general populations, starting with studies from 1966 to 2004 [4]. The review identified 40 studies on FSD in general populations. She examined sexual dysfunction overall and differentiated it into the four major domains: sexual desire disorder, sexual arousal disorder, orgasm disorder, and pain disorders. West et al. encountered two major challenges in the research: (1) the ever-changing definitions of sexual disorders limit a comparison across studies and (2) none of the studies used psychometrically validated instruments to assess dysfunction [4]. This made it impossible to provide an overall prevalence of FSD. West et al. concluded: ‘In all of the research done in this field to date, none of the studies meet the quality criteria set for prevalence studies (i.e., a well-defined representative sample and a validated assessment of female sexual dysfunction)’ [4].

Since 2004, further studies on FSD have been performed around the globe in general populations. Therefore, we see the need for a renewed systematic review of the current literature on the prevalence of FSD in general populations. Through this review, we also intend to identify significant predictors of FSD, as well as the psychometrically validated assessment tools which have been used to assess FSD.

### Previous reviews on FSD in clinical populations

Recent systematic reviews focus on FSD primarily in clinical populations, for example, in patients with diabetes [5], cardiovascular disease [6], psoriasis [7], or depression [8].

In 2013, Pontiroli et al. established that FSD is indeed significantly more frequent among women with diabetes than those without [5]. These were the results of 26 studies which employed the Female Sexual Function Index (FSFI) by Rosen [9] to measure dysfunction. A low FSFI score (indicator of dysfunction) was associated with high body mass index [5].

Another review in 2013 examined sexual dysfunction in male and female patients with cardiovascular disease. Based on 24 studies, Nascimento et al. discovered that among women, all domains of sexual function (desire, arousal, vaginal lubrication, orgasm, sexual dissatisfaction, and pain) were affected by the disease [6]. Furthermore, sexual dysfunction varied according to severity of cardiovascular disease [6].

In 2012, Kurizky et al. compiled a review of studies related to patients (male and female) with psoriasis. A multilingual search of literature between 1966 and 2011 resulted in only eight eligible studies [7]. Results of the review revealed a general decline of sexual function among patients with psoriasis; dysfunction may be influenced by ‘the severity of skin findings, the psychological effects of the condition on the patient, concerns of the sexual partner, and side effects of the medical treatment for psoriasis’ [7]. In addition, results showed that nearly half of all psoriasis patients lamented that healthcare professionals did not sufficiently address their sexual problems [7].

Another recent review from 2012 measured the bidirectional association of depression and sexual dysfunction among men and women [8]. Atlantis and Sullivan pooled odds ratios or relative risks across studies using random-effects meta-analysis models [8]. The analysis confirmed that depression increased the risk of sexual dysfunction and that sexual dysfunction increased the odds of depression [8].

### Previous reviews on FSD in general population samples

We have identified only two systematic reviews that addressed FSD in the general population: Spector and Carey in 1990 [10] and West et al. in 2004 [4].

In Spector and Carey's critical review of 23 studies, the incidence and prevalence of sexual dysfunction among men and women were assessed. Incidence was seldom reported; therefore, the prevalence of dysfunction for men and women was presented. Due to the limited number of community studies, prevalence rates in the domains of FSD varied considerably (female arousal disorder 11%–48%) or were non-existent (no data on the pain disorder vaginismus) [10]. However, determinants such as age, education, socio-economic status, marital status, and gender were found to have an influence sexual dysfunction in men and women [10]. Spector and Carey had three points of critique: (1) populations were not representative, (2) assessment tools varied considerably, and (3) the definitions of dysfunction were inconsistent between studies [10]. Thus, recommendations for future research included ‘stratified samples representative of the general population, the use of psychometrically sound assessment techniques to facilitate interpretation and replication and finally, a common classification system to aid comparison across studies’ [10].

A systematic review by West et al. over 10 years later highlighted similar study limitations as those reported by Spector and Carey. West et al. stated that there still seems to be a lack of data on general populations, since finding a large representative sample is challenging and expensive. West et al. reiterated that the varying definitions of dysfunction as well as the lack of standardized, valid assessment tools present a challenge in comparing prevalence across studies [4]. Although it was not possible to provide an overall prevalence of FSD in general populations, West et al. was able to provide individual prevalence rates of FSD from 40 international studies, illustrating increased interest in FSD between 1990 and 2004. Furthermore, she identified similar predictors of FSD in general populations, e.g., age, education, socio-economic status, and relationship with partner [4]. She also uncovered new determinants such as physical health (both observed and perceived), race/ethnicity, emotional condition, number of premarital partners, religion, sexual orientation, as well as the rigidity of gender roles in a relationship [4].

### Rationale

There is substantial justification for a systematic review of the prevalence and predictors of FSD. First of all, the most recent systematic literature review is 10 years old, beckoning a new systematic review of all currently available literature. Secondly, as indicated by some of West et al.'s determinants of FSD, the predictors of sexual dysfunction may vary over time, in response to cultural shifts, as well as generational and societal norms. Furthermore, within the past few years, first-ever population studies have been performed in countries in which the topic of female sexuality has generally been taboo. Studies on FSD among Iranian [11,12] or Egyptian women [13] may provide a valuable contribution to the global perspective on FSD. Finally, in addition to compiling current prevalence rates, analyzing significant predictors for each domain of FSD will help identify risk populations within the general population.

## Methods/Design

### Protocol and registration

The methods for this systematic review have been developed according to recommendations from the Preferred Reporting Items for Systematic Reviews and Meta-Analyses (PRISMA) statements [14]. This protocol has been registered in the International Prospective Register of Systematic Reviews (PROSPERO): CRD42014009526.

### Objective

This study aims to assess the prevalence and predictors of FSD among various age groups of women in general populations

### Search strategy

We will begin by developing a comprehensive database containing all published studies addressing the prevalence of FSD in general populations. A systematic search of MEDLINE, EMBASE, PsycINFO, and Web of Science will be undertaken. Because our focus is on more recent studies on FSD, we will examine publications from 2000 to 2014. For a list of terms which will be searched, see Additional file 1. The systematic search will be performed in close cooperation with a medical research librarian. Searches will be limited to studies of humans and to peer-reviewed full text articles in English. Duplicates will be removed. Additional citations will be located by searching through conference proceedings (World Meeting on Sexual Health, Congress of the European Society for Sexual Medicine). Finally, reference lists will be searched manually for relevant studies.

### Selection criteria

The population of interest will include adult women in the general population, from menarche to menopause. Cross-sectional, cohort, and case-control studies will be included in this systematic review. The study should report the prevalence of at least one domain of FSD. There will be no geographical limitation on the included studies. Only publications in the English language will be included.

### Study selection

Search results will be imported into Endnote. One reviewer will screen titles and abstracts for their potential relevance. If there is any uncertainty at this stage, the article will remain included until the full text is reviewed. Articles identified through reference lists of included studies and relevant systematic reviews will be considered for inclusion on the basis of their title.

Two reviewers (MEM and MAT) will then assess the full text of all articles identified in the screening process for potential inclusion. Where information pertinent to inclusion criteria is not contained within the article text, the effort will be made to contact the listed corresponding author. Where no reply is received, the article will be excluded. Consensus between the two authors undertaking review of the study will need to be reached before the article is included. Inclusion disagreement will be discussed and resolved by consensus or arbitration by a third investigator (CA). A PRISMA flow chart of the study selection procedure will be prepared, and a log of rejected studies will be maintained.

### Data extraction

Data will be extracted from the included studies using a pre-designed, pilot-tested electronic data form (Microsoft Access). The form has been pilot-tested on ten randomly selected publications on the prevalence and predictors of FSD. Based on the results of the pilot test, the form was revised by the authors. Using the electronic form, one review author (MEM) will extract the data from the included studies and a second author (MAT) will validate the extracted data. Disagreements will be resolved by discussion between the two reviewers; if no agreement can be reached, consensus will be sought through discussions with the third author (CA). Data will be extracted on the following:

1. Publication details: title, journal, author(s), year, city, and country in which the study was conducted, type of publication, and source of funding

2. Design: type of study (cross-sectional, cohort, case-control), aims of study, method of data collection, response rate, recruitment methods, eligibility (inclusion and exclusion criteria), name of assessment tool(s), validation of assessment tool(s)

3. Study participant details: number of persons interviewed or surveyed, population characteristics including age, relationship status, demographic information

4. Data for outcome measures: prevalence of FSD, time period referenced in assessment, significant predictors for each domain of FSD

5. Limitations: selection bias, response bias, information bias, limitations of assessment tool(s) used

### Quality assessment

At the time of the review in 2004, West et al. lacked a tool for assessing the quality of observational studies. However, evaluation of study quality was based on (1) how women were recruited and (2) how the information was obtained. West et al. described that both selection and information bias had an impact on the results of her review. For this review, we will use the Newcastle-Ottawa Scale for the quality assessment of non-randomized studies [15]. Quality scores will be presented in a table.

### Data synthesis

Relevant data extracted from eligible studies will be presented in evidence tables (see Additional files 2 and 3). A narrative synthesis will provide a summary of the prevalence of FSD according to age, as well as the predictors of FSD. Limitations of the studies will be discussed in detail. Implications of the review as well as suggestions for future research will also be provided.

## Discussion

In the past decade, population studies on FSD have increased in number and grown more varied in their cultural setting. The proposed systematic review aims to provide a current overview of the prevalence of FSD in general female populations from various countries, cultures, and age groups in order to allow a better understanding of its effect on women's lives today.

### Strengths and limitations

The strengths of this review include clearly established purpose, as well as a systematic and transparent approach. Our search will be performed in close cooperation with a specialized research librarian with a medical degree; the screening and extraction will be performed cooperatively by two researchers employing pretested, standardized extraction forms. Unlike the previous reviews, this review will provide significant results on the predictors for each domain of FSD. Furthermore, quality assessment of each study will be performed and presented in a table. While the review may include novel studies from a wide variety of cultures, a limitation of the study is its restriction to the English language. Despite a predefined systematic approach, the study will also involve judgments made by review authors, which can result in bias.

### Dissemination

Findings will be disseminated through publication in peer-reviewed journals and conference presentations at relevant conferences.

## Abbreviations

FSD: Female sexual dysfunction.

## Competing interests

The authors declare they have no competing interests in relation to the planned systematic review.

## Authors’ contributions

MEM initiated and conceptualized the study, reviewed previous systematic reviews, and wrote the manuscript. CA advised on background and methodology section and revised the manuscript for important intellectual content. MAT provided insight on epidemiological aspects of the review and helped draft the manuscript. All authors read and approved the final manuscript.

## Supplementary Material

Additional file 1 **Search strategy.** Proposed search terms to be used in the systematic search of literature databases. Click here for file

Additional file 2 **Evidence table.** Proposed table for presenting the extracted data from eligible studies. Click here for file

Additional file 3 **Predictor table.** Significant predictors of female sexual dysfunction. Click here for file
