# Supplementary material for: Prevalence and predictors of female sexual dysfunction: a protocol for a systematic review
Source: Syst Rev. 2014 Jul 11;3:75. doi: 10.1186/2046-4053-3-75 (PMC4108968; doi:10.1186/2046-4053-3-75)
Supplement: Additional file 1 — Search strategy. Proposed search terms to be used in the systematic search of literature databases. [file 2046-4053-3-75-S1.pdf]

Additional file 1 | Search strategy

Proposed Medline Search Terms*

Controlled vocabulary and text/key words
“Sexual Dysfunction, Physiological"[Mesh] OR “Sexual Dysfunctions, Psychological"[Mesh]
"sexual dysfunction*" OR "sexual disorder*" OR "sexual function*" OR "sexual activity" [AllFields]

Synonyms
("orgasm disorder"[Title/Abstract])
("desire disorder" OR sexual interest OR sexual desire OR sexual drive OR sexual aversion OR "hypoactive sexual desire disorder"[Title/Abstract])
("arousal disorder" OR "female sexual arousal disorder" OR sexual stimulation OR sexual excitement OR lubrication[Title/Abstract])
("pain disorder" OR vaginismus OR dyspareunia OR sexual difficult* OR sexual problem* OR "vaginal pain" OR "genital pain" OR sexual discomfort[Title/Abstract])
("sexual satisfaction" OR "sexual dissatisfaction" OR "sexual pleasure" OR "sexual enjoyment"[Title/Abstract])
("sexual distress" OR "sexual stress"[Title/Abstract])

Outcome (prevalence)
epidemiology [MeSH Major Topic]
epidemiologic studies [MeSH Major Topic]
incidence [MeSH Major Topic]
prevalence [MeSH Major Topic]
(incidence OR prevalence OR occur* OR frequenc* OR proportion* OR rate* OR number* OR percent* [Title/Abstract])

Population terms
female OR women OR woman [AllFields]
"general population" OR "population study" OR survey OR "representative sample" OR "representative population" OR sample OR "community study" OR community

Filters activated
Publication date from 01.01.2000 to current date in 2014, Humans, English language

*Search terms and strategy will be adapted to database: Embase, PsycInfo, Web of Science
