# Supplementary material for: Prevalence and predictors of female sexual dysfunction: a protocol for a systematic review
Source: Syst Rev. 2014 Jul 11;3:75. doi: 10.1186/2046-4053-3-75 (PMC4108968; doi:10.1186/2046-4053-3-75)
Supplement: Additional file 2 — Evidence table. Proposed table for presenting the extracted data from eligible studies. [file 2046-4053-3-75-S2.pdf]

Additional file 2 | Evidence table

Author(s), year, [illegible]untry	Study design, method, response rate	Assessment tool, validation	Recruitment methods, eligibility	Population characteristics	Prevalence rates	Time period referenced	Newcastle Ottawa Scale results	Limitations of study, comments
