# Supplementary material for: Prevalence and predictors of female sexual dysfunction: a protocol for a systematic review
Source: Syst Rev. 2014 Jul 11;3:75. doi: 10.1186/2046-4053-3-75 (PMC4108968; doi:10.1186/2046-4053-3-75)
Supplement: Additional file 3 — Predictor table. Significant predictors of female sexual dysfunction. [file 2046-4053-3-75-S3.pdf]

Additional File 3 | Predictor table

Significant predictors of female sexual dysfunction

	Predictor 1	Predictor 2	Predictor 3	Predictor 4	Predictor 5	Predictor 6	Predictor 7	Predictor 8
**Disorder 1**								
Study A								
Study B								
…								
**Disorder 2**								
Study C								
Study D								
…								
**Disorder 3**								
Study E								
Study F								
…								

– negative association, protective factor

\+ positive association, risk factor

[1] univariate/crude, otherwise multivariate/adjusted analysis
